# Call to action for an invigorated drive to scale up TB prevention

**DOI:** 10.5588/ijtld.21.0421

**Published:** 2021-09-01

**Authors:** T. Kasaeva, A. Kanchar, M. H. Dias, D. Falzon, M. Zignol, A. Pablos-Mendez

**Affiliations:** 1 Global Tuberculosis Programme, WHO, Geneva, Switzerland; 2 Division of General Medicine, Columbia University Medical Center, New York, NY, USA

On 16 June 2021, the WHO convened a high-level event to shine a spotlight on the pressing need to ramp up TB prevention worldwide.^1^ The event, “A global drive to scale up TB prevention”, echoed the commitments and ambitious goals towards ending TB established by heads of States at the United Nations High-Level Meeting (UN-HLM) on TB in 2018.^2^ These include targets to provide TB preventive treatment (TPT) to at least 6 million people living with HIV, as well as 4 million contacts under 5 years of age and 20 million older contacts by 2022. While the target for people living with HIV is expected to have been achieved in 2020, out of the 24 million contacts more than 22 million have yet to be reached.

TB remains one of the top infectious killers worldwide and a major cause of disease.^3^ One quarter of the world’s population is estimated to be infected with *Mycobacterium tuberculosis*, a large reservoir that could fuel TB incidence in the coming decades.^4^ Only treating people with active TB disease is thus insufficient to end TB. While TB preventive measures are, therefore, necessary to save lives, prevent long-term disability and alleviate suffering, they are often neglected. Providing TPT to reach the UN-HLM targets will require a massive scale-up of effort and investment. Offering TPT solely to contacts of people with TB who present to health services will not bridge the gap of 22 million by 2022. Active case-finding is needed to detect TB in populations with poor access to health services who are unlikely to seek timely care and to offer TPT to their contacts.

With 18 months left to achieve these goals, WHO and its partners are calling on governments and other stakeholders to keep the promises they have made and urgently accelerate coverage of TPT for those in need. This is made even more pressing by the COVID-19 pandemic, a crisis that has profoundly disrupted TB care, supply chains and other programme activities over the past 18 months and reversed gains made in recent years in lowering TB incidence and mortality.^5^ For this reason, the UN Secretary General in his 2020 Progress Report on TB called for prioritising and dramatically scaling-up access to TPT and stronger multisectoral action in the context of the pandemic.^6^

At the opening of the high-level event, Dr Tedros Adhanom Ghebreyesus, WHO Director-General, urged governments and partners to build upon recent successes in the scale-up of TPT among people living with HIV, and to accelerate the drive to bring many more millions of people onto TPT, particularly children and other contacts of people with TB. He also underlined the negative impact of the COVID-19 pandemic on TB prevention and care. During the event, a TB survivor made a moving appeal for scaling-up access to TPT so that people and families are spared the suffering that she endured. Ministers of Health from Brazil, India, Indonesia, Nigeria, Pakistan, the Philippines and the Russian Federation, major donors, representatives of civil society and technical agencies also reported their work, vision and commitments to step up TB preventive action (see Figure).

**Figure:**
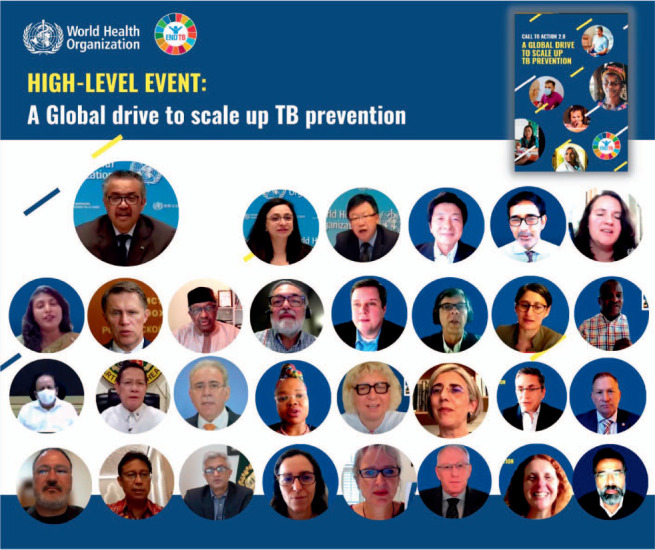
Global leaders urge for a radical drive to find and prevent TB in the next 18 months.

As part of the event, the WHO, governments, civil society and other partners launched a ‘Call to Action’ for broader access to TPT for those in need.^7^ The call stressed the need for a set of key actions to achieve its goals (see Table), and urged countries and donors in the next 18 months to 1) strengthen and finance TB preventive interventions adequately, as a sound investment and in recognition of TB prevention as a human right and entitlement under the rights to health. Achieving the UN-HLM targets for TPT alone requires an annual budgetary increase of the order of one third of the USD6.5 billion currently allocated globally for TB activities each year. This would still be well below the USD13 billion considered necessary to fund all TB programme components adequately each year worldwide; 2) to expand outreach by giving TPT to an average of three people in contact with each person with bacteriologically confirmed TB; and 3) to fully activate systematic screening to find more people with TB and in need of TPT in order to achieve the UN-HLM targets on TPT. In addition to the people with TB presenting to healthcare services, 6 million more need to be found by active case-finding.

**Table:** Key actions to rapidly scale up TB preventive treatment and screening worldwide

Strengthen TB case-finding in household contacts of all ages and other high-risk groups, such as residents of informal settlements, to accelerate progress, in synergy with efforts for COVID-19 pandemic mitigation.
Embrace and increase access to WHO-recommended diagnostic technologies such as digital radiography, computer-aided detection software, molecular rapid diagnostic testing and tests of TB infection.
Rapidly reinforce programme capacity to ensure affordable access to WHO-recommended shorter TB preventive treatment regimens for people of all ages.
Apply successful strategies in the large-scale roll-out of TB preventive treatment among people living with HIV to increase coverage of all populations in need.
Launch a large-scale communication and advocacy campaign to create demand for TB preventive treatment, and to enhance its acceptability among those who need it, as well as healthcare providers.
Continue to strengthen all key measures that influence prevention, including improved case-finding in primary care, TB infection prevention and control, ending stigma, poverty alleviation, social protection and universal health coverage.
Partner with and mobilise communities to generate demand for TB preventive treatment and other prevention services, and to strengthen monitoring and delivery systems.
Support research and innovation on TB prevention, especially on vaccine development.

The ‘Call to Action’ was developed through a broad-based stakeholder consultation and was endorsed by the WHO Civil Society Taskforce and 18 major donors, implementing partners and professional bodies, including Aurum Institute (Johannesburg, South Africa), American Thoracic Society (New York, NY, USA), Elizabeth Glaser Pediatric Aids Foundation (Washington DC, USA), European Respiratory Society (Lausanne, Switzerland), FIND (Geneva, Switzerland), The Global Fund (Geneva, Switzerland), International AIDS Society (Geneva, Switzerland), KNCV Tuberculosis Foundation (The Hague, The Netherlands), Médecins Sans Frontières (Paris, France), Sentinel Project (Boston, MA, USA), Stop TB Partnership (Geneva, Switzerland), Treatment Action Group (New York, NY, USA), International Union Against Tuberculosis and Lung Disease (Paris, France), UNICEF (New York, NY, USA), Unitaid (Geneva, Switzerland), USAID (Washington DC, USA), US Centre for Disease Control and Prevention (Atlanta, GA, USA) and the US President’s Emergency Plan for AIDS Relief (Washington DC, USA). The ‘Call to Action’ was also fully supported by the Strategic and Technical Advisory Group for TB, that guides the work of the WHO on TB, during its annual meeting held the week after the event.^8^ Other institutions have also stepped forward since to support the initiative.

A swift translation of the ‘Call to Action’ into national programmatic action will be needed to precipitate radical change in the coming months. The WHO is engaging with countries and partners to support this process, seeking enablers and synergies to overcome operational barriers. Without the imminent prospect of an immunising vaccine that can be quickly scaled up, TPT and screening remain indispensable components of any comprehensive TB strategy in both high and low TB burden settings. However, a global drive in this direction needs to be accompanied by sustained action in other areas critical to achieving the End TB Strategy targets beyond 2022, such as TB infection prevention and control, social protection and universal health coverage.^9^,^10^ Continued implementation of new technologies at a much more rapid pace will be needed to achieve these changes and to create more resilient health systems and primary care services. Emerging technologies will also need to be implemented as supported by evidence.
